# α-MSH Analogue Attenuates Blood Pressure Elevation in DOCA-Salt Hypertensive Mice

**DOI:** 10.1371/journal.pone.0072857

**Published:** 2013-08-16

**Authors:** Petteri Rinne, Anna-Maija Penttinen, Wendy Nordlund, Markku Ahotupa, Eriika Savontaus

**Affiliations:** 1 Department of Pharmacology, Drug Development and Therapeutics, and Turku Center for Disease Modeling, University of Turku, Turku, Finland; 2 Department of Biochemistry and Food Chemistry, University of Turku, Turku, Finland; 3 Unit of Clinical Pharmacology, Turku University Hospital, Turku, Finland; National Institute of Nutrition, India

## Abstract

Melanocyte-stimulating hormones, α-, β- and γ-MSH, regulate important physiological functions including energy homeostasis, inflammation and sodium metabolism. Previous studies have shown that α-MSH increases sodium excretion and promotes vascular function in rodents, but it is unexplored whether these characteristics of α-MSH could translate into therapeutic benefits in the treatment of hypertension. Therefore, we first assessed the diuretic and natriuretic properties of the stable α-MSH analogue [Nle^4^, D-Phe^7^]-α-MSH (NDP-α-MSH) and investigated whether it has protective effects in deoxycorticosterone acetate (DOCA)-salt hypertensive mice. Adult male C57Bl/6N mice were subjected to DOCA-salt treatment and randomized to receive intraperitoneal injections of either saline as vehicle or NDP-α-MSH (0.3 mg/kg/day for 14 days) starting 7 days after the DOCA-salt treatment. Systemic hemodynamics, serum and urine electrolytes, and oxidative stress markers were assessed in control sham-operated and DOCA-salt mice. NDP-α-MSH elicited marked diuretic and natriuretic responses that were reversible with the MC3/4 receptor antagonist SHU9119. Chronic NDP-α-MSH treatment attenuated blood pressure elevation in DOCA-salt mice without affecting the blood pressure of normotensive control animals. Owing to the enhanced sodium excretion, NDP-α-MSH-treated mice were protected from DOCA-salt-induced hypernatremia. DOCA-salt treatment mildly increased oxidative stress at the tissue level, but NDP-α-MSH had no significant effects on the oxidative stress markers. In conclusion, treatment with NDP-α-MSH increases urinary sodium excretion and protects against DOCA-salt-induced hypertension. These findings point to the potential future use of α-MSH analogues in the treatment of hypertension.

## Introduction

Melanocyte-stimulating hormones (α-, β- and γ-MSH) and adrenocorticotropic hormone or melanocortins are a family of peptides that are proteolytically cleaved from a common precursor molecule known as pro-opiomelanocortin. They regulate a multitude of physiological functions including energy homeostasis, electrolyte balance and inflammatory responses. Early studies demonstrated that acute administration of α-MSH or β-MSH increases urinary sodium excretion in rodents, indicating a natriuretic property for these peptides [1,2]. Additional research provided evidence that γ-MSH is also a natriuretic peptide and seems to be involved in the physiological regulation of sodium balance [3,4]. The natriuretic action of melanocortins is thought to be mediated, at least in part, by the melanocortin 3 receptor (MC3-R) expressed in the kidney [4]. However, it is an open question whether pharmacological targeting of the MC3-R could have therapeutic efficacy in experimental models of hypertension.

Aside from possessing a natriuretic property, we have recently demonstrated that α-MSH, by interacting with endothelial MC1 receptors, regulates the local control of blood vessel tone by enhancing nitric oxide (NO) availability and NO-dependent vasodilatation [5]. In keeping with the vascular protective role of α-MSH, pharmacological treatment with the stable α-MSH analogue [Nle^4^, D-Phe^7^]-α-MSH (NDP-α-MSH) showed promising effects in animal models of vascular dysfunction. Given this, along with other studies showing that NDP-α-MSH has protective effects in cardiovascular disease models such as myocardial infarct [6–8], we hypothesized that NDP-α-MSH treatment, through its vascular effects and a probable natriuretic property, translates into therapeutic benefits in an animal model of hypertension.

In the present study, we first assessed whether NDP-α-MSH elicits diuretic and natriuretic responses in mice. Having identified marked diuretic and natriuretic properties for this compound, we sought to determine whether chronic treatment with NDP-α-MSH protects against deoxycorticosterone acetate and salt (DOCA-salt)-induced hypertension. We chose to use NDP-α-MSH instead of γ-MSH or its analogues since it has high affinity for MC1 and MC3 receptors and would therefore efficiently target both these MC-R pathways. Since oxidative stress is known to contribute to the development of hypertension [9], combined with the concept that α-MSH can suppress oxidative damage in cardiovascular disease models [6,10–12], we aimed to evaluate the effects of NDP-α-MSH treatment on oxidative stress markers in DOCA-salt mice.

## Materials and Methods

### Animals

Experiments were performed on adult male C57Bl/6N mice. Animals were housed on a 12 h light/dark cycle and fed *ad libitum* a regular chow diet. All experiments were approved by the national Animal Experiment Board in Finland (Permit Number: ESAVI-438/04.10.03/2012) and conducted in accordance with the Directive 2010/63/EU of the European Parliament.

### Drug treatments and measurements of diuresis

NDP-α-MSH (obtained from Sigma-Aldrich) was given as an intraperitoneal (i.p.) injection in a dose range of 1-300 µg/kg. Acute diuresis, occurring within the first 4 hours after the injection, was evaluated based on a change in body weight. Body composition was analyzed by quantitative NMR scanning (EchoMRI-700, Echo Medical Systems, TX, USA) to demonstrate the validity of body weight monitoring as a method to assess the diuretic response to NDP-α-MSH administration. Diuretic effect was also assessed by collecting and measuring 24-h urine output. To evaluate the dependence of the diuretic responses on MC receptor activation, mice were pretreated with the selective MC3/4-R antagonist SHU9119 (1 mg/kg i.p.) given 30 minutes prior to NDP-α-MSH (0.3 mg/kg) administration. Control saline (0.9% NaCl) and drug injections were given in a volume of 10 µl/g body weight.

### DOCA-salt hypertension model

To investigate the effects of NDP-α-MSH in hypertensive animals, we subjected 4-month-old mice to DOCA-salt treatment. A slow-release (21-day) 50 mg DOCA pellet (Innovative Research of America, FL, USA) was implanted through a mid-scapular incision under isoflurane-anesthesia. DOCA-salt animals received 1% NaCl in drinking water starting with the first day of DOCA-treatment. Control animals were sham-operated and received normal drinking water. After 7 days of DOCA-treatment, control and DOCA-salt mice were randomized to receive daily i.p. injections of either saline or NDP-α-MSH (0.3 mg/kg) for the following 14 days.

### Arterial pressure measurements

Blood pressure was measured in conscious, unrestrained mice using a radiotelemetry system (TA11PA-C10 and Dataquest software, Data Sciences International, NM, USA) as previously described [13,14] or by a noninvasive tail-cuff method (TSE Systems, Germany) in conscious, restrained mice. For tail-cuff measurements, the mice were trained for at least 2 consecutive days before the actual data collection. In each recording session, the mice were placed on a heated pad (35 °C) and allowed to settle for at least 5 min before data acquisition. The average of 10 readings from each mouse was recorded.

### Urine analyses

Twenty-four-hour urine samples were collected in metabolic cages. In the DOCA-salt experiment, samples were collected at the end of the 14-day NDP-α-MSH treatment. Urine sodium and potassium concentrations were measured by flame photometry. Urinary creatinine, NO_x_ (nitrate+nitrite), cGMP and 8-isoprostane concentrations were determined with commercially available assay kits (Cayman Chemicals, MI, USA).

### In situ detection of vascular superoxide production

Frozen mouse aortae were cut into 10 μm thick transverse sections and placed on a glass slide. Dihydroethidium (5 μM, Molecular Probes, OR, USA) was topically applied onto each section and incubated for 30 min at 37 °C to reveal the presence of superoxide anions (O_2_^●−^) by fluorescence microscopy.

### Measurements of oxidation products

For oxidative stress analyses, tissue samples were homogenized in 0.1 M Tris buffer with 1 mM EDTA (pH 7.4). For the measurement of conjugated dienes (CD), lipids were extracted from tissue homogenates using a 2:1 mixture of chloroform and methanol, dried under nitrogen atmosphere and redissolved in cyclohexane. CDs were then analysed spectrophotometrically at 234 nm. 8-isoprostanes were determined by STAT-8-Isoprostane EIA kit (Cayman Chemicals).

### Statistics

All data are expressed as means ± SEM. Dose-responses were analyzed using one-way analysis of variance (ANOVA) followed by Tukey’s multiple comparison tests. Comparisons among three or more groups were made by one-way ANOVA followed by Bonferroni *post hoc* tests. Follow-up data on blood pressure was analyzed by two-way ANOVA and Bonferroni *post hoc* tests. For two independent factors, two-way ANOVA was used. A two-tailed P value of less than 0.05 was considered statistically significant.

## Results

### Administration of NDP-α-MSH induces diuresis and natriuresis through the activation of MC3/4 receptor pathways

**Figure 1:**
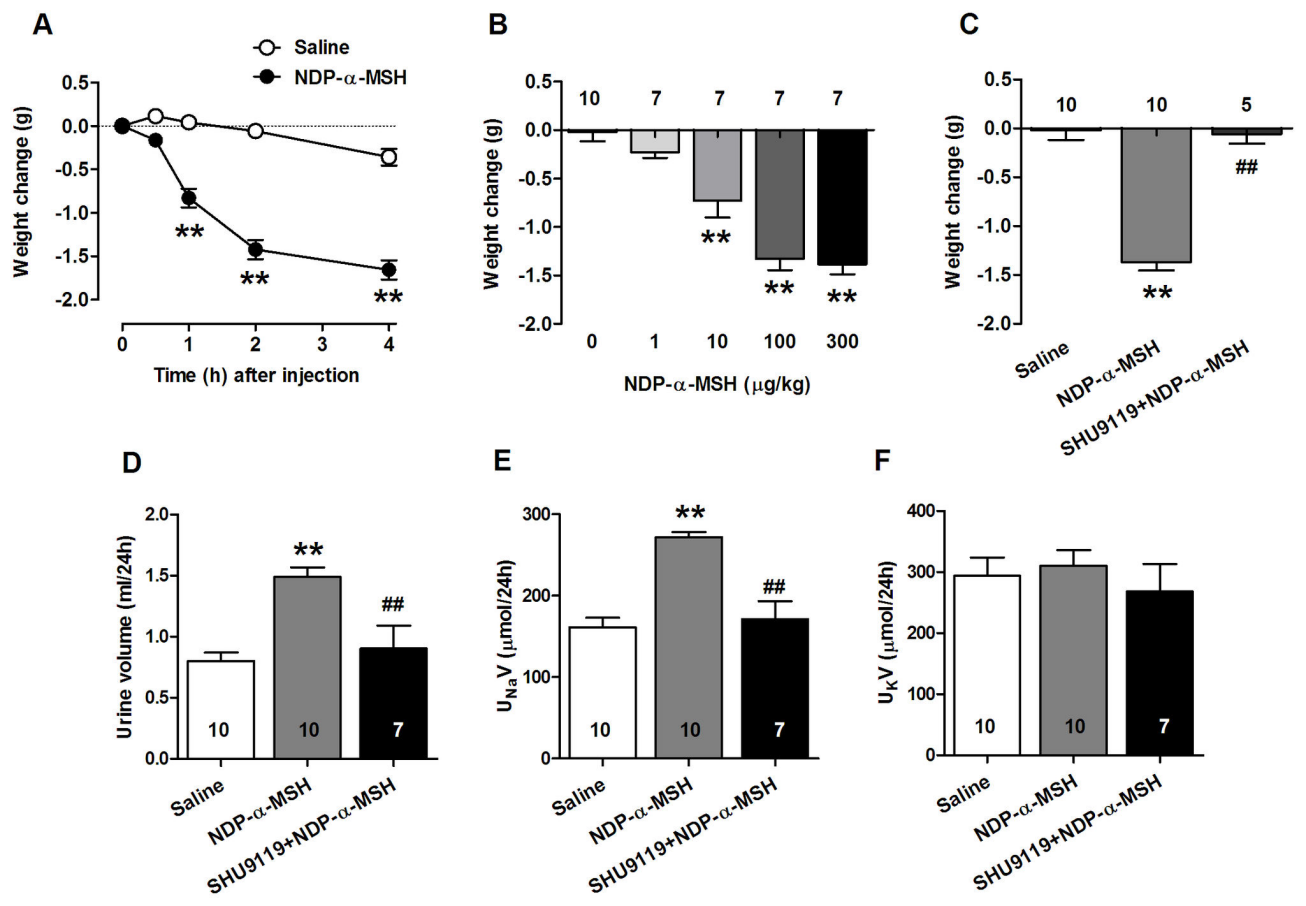
NDP-α-MSH induces diuretic and natriuretic responses that are blocked with the MC3/4 receptor antagonist SHU9119. (**A**) Time course of weight change after an intraperitoneal (i.p.) injection of saline and NDP-α-MSH (0.3 mg/kg). The values are based on a change from pretreatment weight. n = 7 per group. (**B**) Dose-dependent effects of NDP-α-MSH on body weight. Mice were weighed 2 hours after the injection. (**C**) Effect of a selective MC3/4-R antagonist (SHU9119) on NDP-α-MSH-induced diuresis. SHU9119 (1 mg/kg i.p) was given 30 min prior to administration of NDP-α-MSH (0.3 mg/kg i.p.). (**D**–**F**) 24-hour urine volume and urinary excretion of sodium (U N_a_V) and potassium (U _K_V) in control, NDP-α-MSH-treated and SHU9119-pretreated mice. The number of mice analyzed is shown in the graphs. Data are mean ± SEM. ** *P* < 0.01 versus saline, # # *P* < 0.01 versus NDP-α-MSH.

NDP-α-MSH induced a marked diuretic effect as evidenced by a change in body weight (Figure **1A**). Body composition analysis revealed that the NDP-α-MSH-evoked weight reduction is exclusively attributable to a loss in total body water (Figure **S1**). The reduction in body weight occurred in a dose-dependent manner (Figure **1B**). Pretreatment with the MC3/4-R receptor antagonist SHU9119 prevented the NDP-α-MSH-induced diuresis (Figure **1C**). Supporting these findings, 24-h urine volume was increased by NDP-α-MSH administration (Figure **1D**). Furthermore, NDP-α-MSH markedly increased urinary excretion of sodium (Figure **1E**), indicating natriuretic activity. It is noteworthy that NDP-α-MSH administration did not cause kaliuresis despite the profound diuresis and natriuresis (Figure **1F**). Pretreatment with SHU9119 inhibited completely NDP-α-MSH-induced diuresis as well as natriuresis (Figure **1, D and E**).

### NDP-α-MSH attenuates hypertension in DOCA-salt mice

**Figure 2:**
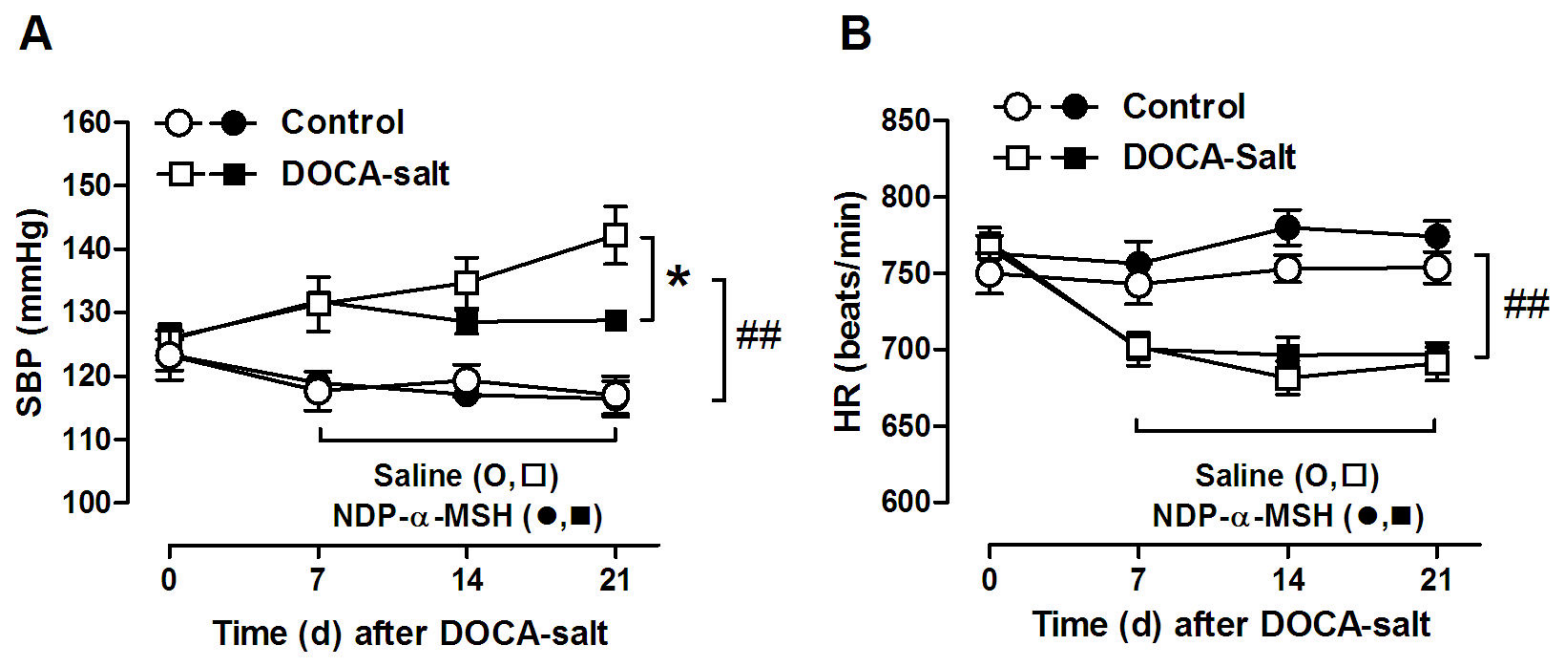
NDP-α-MSH treatment prevents the elevation of blood pressure in DOCA-salt mice. Systolic blood pressure (**A**) and heart rate (**B**) in control and DOCA-salt treated mice measured by a tail cuff method. After 7 days on the control and DOCA-salt treatments mice were randomized to daily i.p. injections of either saline or NDP-α-MSH (0.3 mg/kg). Data are mean ± SEM of six mice per group. * *P* < 0.05 versus saline-treated DOCA-salt mice, # # *P* < 0.01 versus control mice.

**Figure 3:**
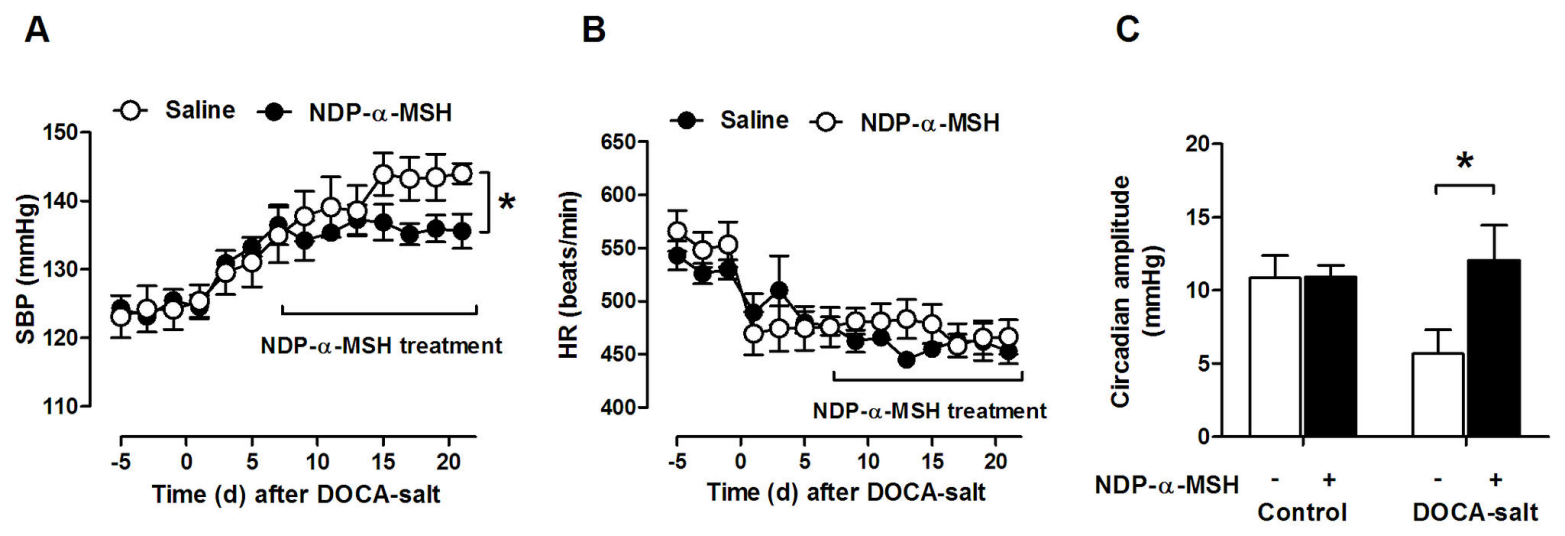
Telemetric measurements of blood pressure in saline- and NDP-α-MSH-treated DOCA-salt mice. Systolic blood pressure (**A**) and heart rate (**B**) in control and DOCA-salt treated mice measured by radiotelemetry. Data represent 24 h mean values of SBP and HR. (**C**) Circadian amplitude, the difference between night- and day-time SBP, at baseline and at day 21 after the beginning of DOCA-salt treatment. * *P* < 0.05 versus saline-treated mice. Data are mean ± SEM of five mice per group.

We next sought to determine whether NDP-α-MSH has any therapeutic efficacy in systemic hypertension. Daily treatment with NDP-α-MSH did not affect body weight change over the 14-day treatment period in DOCA-salt mice (Saline -0.5±0.2 g, NDP-α-MSH 0.2±0.4 g, *P*=0.28), when final body weight was monitored 24 hours after the last injection and compared to the starting weight. Nevertheless, the final dose of NDP-α-MSH caused an acute decrease (-1.4±0.1 g, *P*<0.001 versus saline-treated) in the body weight of DOCA-salt mice which was restored 24 hours after the injection (-0.1±0.2 g, *P*=0.54 versus saline-treated), reflecting a persistent diuretic response during chronic administration of NDP-α-MSH. Notably, NDP-α-MSH was able to restrain the progression of hypertension in DOCA-salt mice without affecting the blood pressure (BP) of normotensive control animals (Figure **2A**). The effect of NDP-α-MSH on BP control was observed in two separate cohorts of mice using two different measurement techniques; tail-cuff plethysmography (Figure **2A**) and radiotelemetry (Figure **3A**). Exposure to DOCA-salt reduced heart rate (HR), but saline- and NDP-α-MSH-treated DOCA-mice showed similar HR (Figures **2B** and **3B**). Interestingly, NDP-α-MSH-treated mice still showed the physiological depression of day-time BP values after 21 days on DOCA-salt, and thus, they displayed an increased circadian BP amplitude compared to saline-treated mice (Figure **3C**).

### NDP-α-MSH protects against DOCA-salt-induced hypernatremia

**Figure 4:**
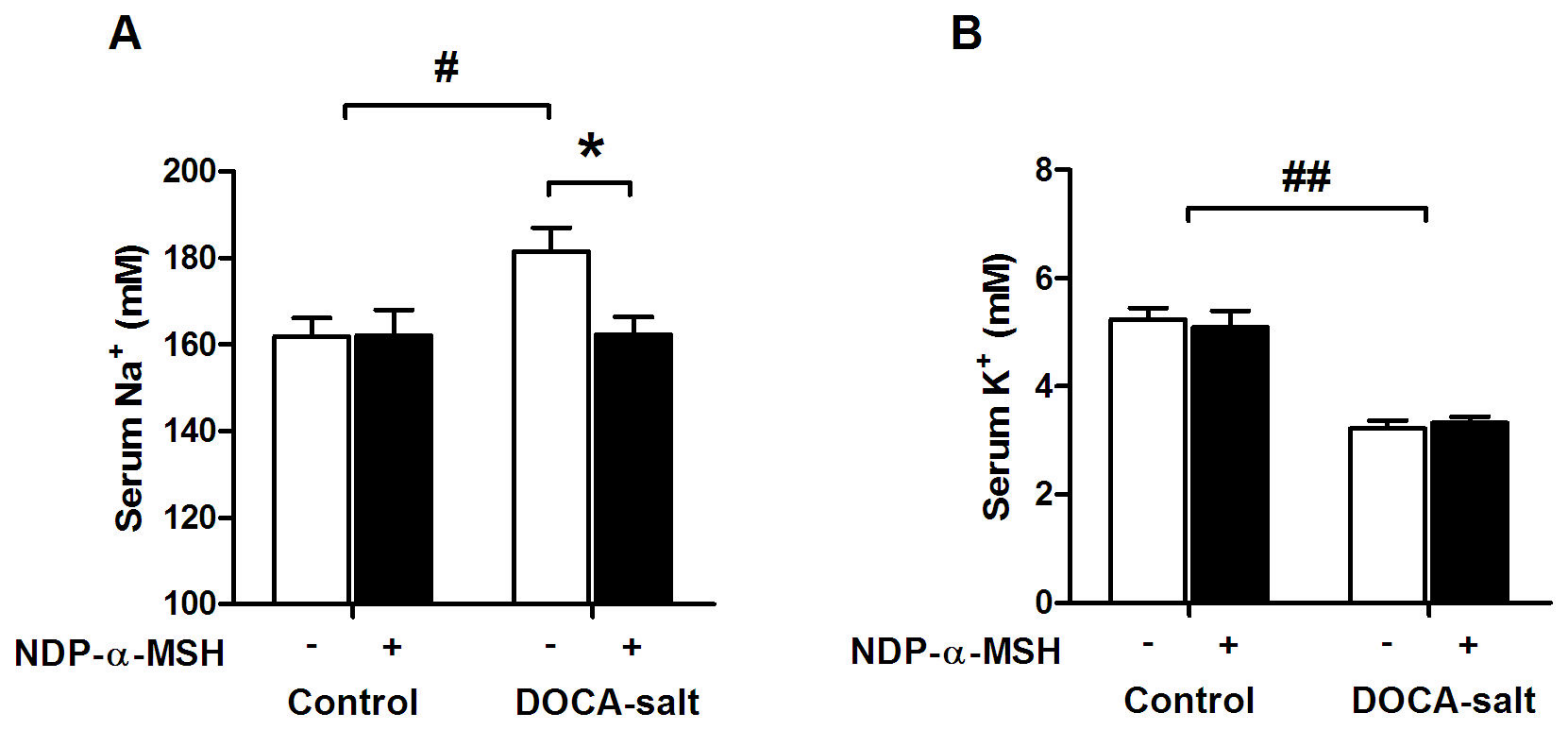
NDP-α-MSH treatment protects against DOCA-salt-induced hypernatremia. Serum sodium (**A**) and potassium (**B**) concentrations in control and DOCA-salt mice. Data are mean ± SEM. Number of mice analyzed is given in Table 1. * *P* < 0.05 versus saline-treated DOCA-salt mice, # *P* < 0.05 and # # *P* < 0.01 versus control mice.

Table **1** describes the effects of NDP-α-MSH-treatment on electrolyte balance in control and DOCA-salt mice. Water intake and urine volume were markedly increased after the DOCA-salt challenge, but NDP-α-MSH caused no significant effects on these parameters. However, NDP-α-MSH-treatment increased urinary sodium excretion in control and DOCA-salt mice (Table **1**). Owing to the enhanced sodium excretion, NDP-α-MSH-treated mice seemed to be protected from DOCA-salt induced hypernatremia (Figure **4A**). DOCA-salt challenge was also associated with hypokalemia caused by excess potassium loss into urine (Table **1**), but NDP-α-MSH-treatment was not able to prevent the disruption of potassium homeostasis (Figure **4B**). Furthermore, no differences were noted in heart or kidney weights between saline- and NDP-α-MSH-treated mice (Table **1**).

**Table 1:** Effects of NDP-α-MSH treatment on body fluid and electrolyte balance, and organ weights in control and DOCA-salt mice.

	**Control**		**DOCA-salt**	
	**Saline**	**NDP-α-MSH**	**Saline**	**NDP-α-MSH**
	*n =7*	*n =7*	*n = 12*	*n = 11*
Body weight (g)	35.0 ± 1.8	34.4 ± 1.6	34.3 ± 0.6	34.5 ± 0.9
**Renal function**				
Water intake (ml/24h)	5.4 ± 0.3	6.0 ± 0.3	19.7 ± 2.9^#^	21.8 ± 2.2^#^
Urine volume (ml/24h)	1.4 ± 0.3	1.9 ± 0.2	12.3 ± 2.3^#^	16.0 ± 1.9^#^
U_Crea_V (mg/24h)	1.04 ± 0.16	1.20 ± 0.15	1.06 ± 0.06	1.09 ± 0.08
U_Na_V (mmol/24h)	0.29 ± 0.05	0.39 ± 0.03*	2.19 ± 0.35^#^	3.30 ± 0.30* #
U_K_V (mmol/24h)	0.53 ± 0.06	0.53 ± 0.08	1.10 ± 0.07^#^	1.30 ± 0.09^#^
**Cardiovascular organs**				
Heart (mg)	172 ± 6	163 ± 3	173 ± 11	161 ± 9
Heart/BW (mg/kg)	5.0 ± 0.2	4.8 ± 0.3	5.1 ± 0.3	4.7 ± 0.2
Kidney (mg)	218 ± 6	201 ± 5	305 ± 20^#^	289 ± 12^#^
Kidney/BW (mg/kg)	6.3 ± 0.3	5.9 ± 0.2	8.9 ± 0.5^#^	8.4 ± 0.3^#^

All measurements are means ± SEM. U_Crea_V, urinary creatinine excretion; U_Na_V, urinary Na^+^ excretion; U_K_V, urinary K^+^ excretion; BW, body weight. Two-way effects by ANOVA: * *P* < 0.05 vs. saline-treated mice; ^#^ *P* < 0.05 vs. control mice.

### Effects of NDP-α-MSH-treatment on NO and cGMP excretion, and oxidative stress markers

**Figure 5:**
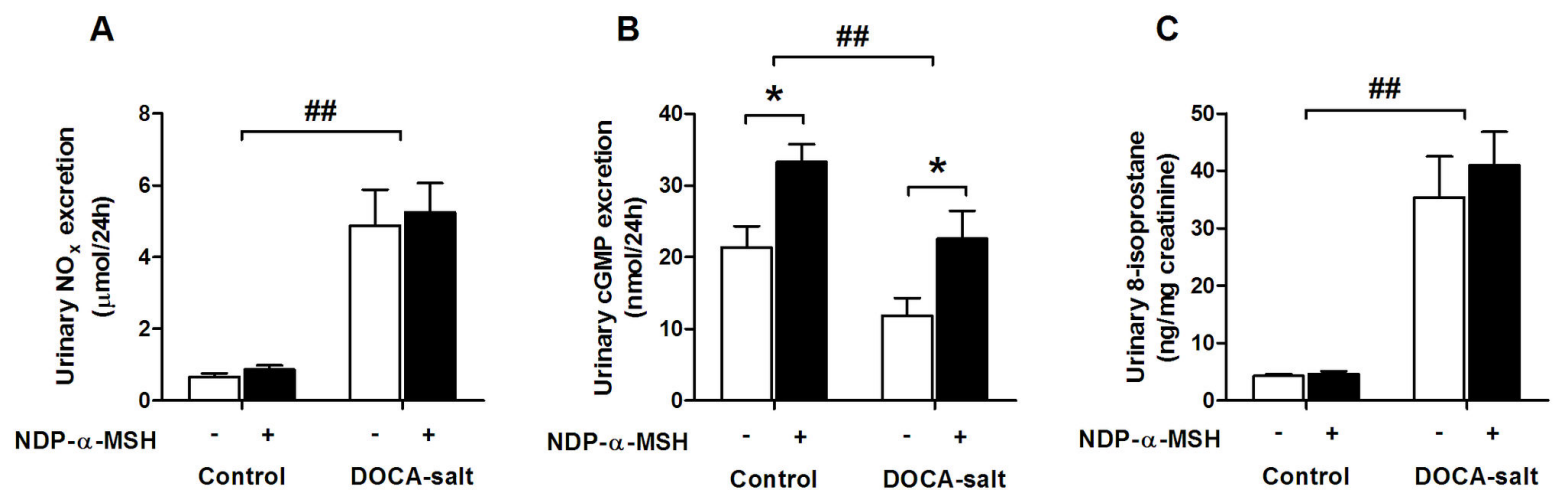
NDP-α-MSH treatment increases urinary excretion of cGMP. Urinary excretion of NO metabolites (**A**), cGMP (**B**) and 8-isoprostane (**C**) in control and DOCA-salt mice. Number of mice analyzed is given in Table 1. * *P* < 0.05 versus saline group, # # *P* < 0.01 versus control mice.

Exposure to DOCA-salt resulted in increased urinary excretion of NO metabolites and reduced excretion of cGMP (Figure **5** **, A and B**). Despite the lack of effect on NO levels, NDP-α-MSH-treatment increased urinary excretion of cGMP in both control and DOCA-salt mice (Figure **5B**). Urinary 8-isoprostane excretion, a marker of oxidative stress, was significantly increased by DOCA-salt-challenge but was unaffected by NDP-α-MSH-treatment (Figure **5C**). To evaluate reactive oxygen species (ROS) generation at the vascular level, we incubated aortic sections with the superoxide indicator dihydroethidium. Vascular ROS production was slightly increased under hypertensive conditions but no effect was noted for NDP-α-MSH in this regard (Figure **S2**). Furthermore, DOCA-salt mice showed reduced 8-isoprostane and unaltered CD levels in the heart and kidney (Figure **S3**). NDP-α-MSH treatment had no effect on these parameters.

## Discussion

The interest in melanocortins and their biology has grown over the years as it has become evident that melanocortins regulate a variety of physiological functions and seem to provide protective effects in experimental disease models encompassing from acute nephropathies to severe ischemic conditions such as myocardial infarct [7,11,12]. The established roles of melanocortins in sodium metabolism and vascular homeostasis prompted us to investigate whether melanocortin treatment could have beneficial effects also in a hypertensive disease state.

In the present study, we observed that the stable analogue of α-MSH induced diuresis and natriuresis, which were evident also during repeated administration. Natriuretic activity, in particular, is a long-known feature of melanocortins, but its exact mechanism is still unclear [1,2,4]. Early work demonstrated that acute administration of α-MSH or β-MSH increases urinary sodium and potassium excretion without producing any change in urine output. If anything, the effect on urine output seemed to be antidiuretic. Thus, our findings shed important new light on the overall effects of α-MSH-like compounds on water and electrolyte excretion. In contrast to previous findings, we found that the potent α-MSH analogue induced diuretic and natriuretic responses without affecting renal potassium handling.

According to current understanding, the natriuretic action of melanocortins is mediated by renal MC3 receptors. Although there are conflicting views about the expression of melanocortin receptor subtypes in the kidney [12,15], supportive evidence for the MC3-R based mechanism came from studies showing that protein for MC3-R is present in the rat kidney and that the expression of MC3-R becomes upregulated when rats are fed with a high sodium diet [16,17]. Furthermore, these studies demonstrated that γ-MSH, which is the natural ligand for the MC3-R, stimulates cAMP formation in cultured inner medullary collecting duct cells. The cAMP response was prevented by SHU9119. These data, combined with the finding that γ-MSH-induced natriuresis was abolished by pretreatment with SHU9119 or the selective MC3-R antagonist SHU9005 [18], provide compelling evidence that functional MC3 receptors are present in the kidney and that they are responsible for mediating the natriuretic action of melanocortins. The present finding that the diuretic and natriuretic actions of NDP-α-MSH were reversible with SHU9119 lends support to this concept. Controversy still remains concerning the role of renal nerves in the natriuretic response, since renal denervation was previously found to prevent the natriuretic response to γ-MSH infusion [19], but Cope et al did not observe such an effect [16]. Although the renal MC3 receptors seem to play a pivotal role, the exact mechanism and site of action of melanocortin-evoked natriuresis are still unknown. Whatever the underlying mechanism, it is important to note that melanocortins and their potent analogues elicit significant and persistent diuretic and natriuretic responses.

Excessive sodium accumulation contributes to the pathogenesis of the DOCA-salt model, indicating a volume-dependence of the hypertension [20]. Against this background, we found it intriguing to investigate whether the diuretic and natriuretic actions of NDP-α-MSH could relieve water and sodium retention and thereby also attenuate hypertension in the DOCA-salt model. Although it did not reverse pre-existing hypertension, we observed that treatment with NDP-α-MSH was able to prevent further elevation of blood pressure and to normalize the circadian blood pressure rhythm in DOCA-salt hypertensive mice. However, the latter effect might reflect the relatively short duration of action of the pharmacological treatment, since the half-life of parenterally administered NDP-α-MSH during the elimination phase is only a few hours [21]. In the present study, NDP-α-MSH was administered during day-time, which might explain why NDP-α-MSH-treated mice showed the normal physiological depression of daytime blood pressure. Accordingly, longer-acting α-MSH analogues or modified dosage regimens could provide further blood pressure control in experimental hypertension. Nevertheless, the present treatment approach seemed to restore sodium balance in DOCA-salt hypertension, since NDP-α-MSH-treated mice were protected from the hypernatremia. It needs to be acknowledged that periodic renal function data would have provided a clearer picture of sodium retention and its development in the DOCA-salt mice, and allowed to assess the therapeutic effect of NDP-α-MSH on water and sodium balance in more detail. The fact that NDP-α-MSH was not able to prevent the appearance of hypokalemia suggests that add-on therapy with a potassium-sparing diuretic and/or potassium supplements would be needed to achieve an improved potassium balance. Collectively, it is noteworthy that NDP-α-MSH treatment may provide therapeutic benefits in the management of hypertension due to its diuretic and natriuretic effects, considering that diuretics are still a cornerstone in the treatment of high blood pressure.

Although eliciting a blood pressure lowering effect in DOCA-salt hypertensive mice, it needs to be taken into account that NDP-α-MSH and other similar compounds acting at the MC4 receptor may potentially cause acute elevations in blood pressure and heart rate. A number of human and animal studies have demonstrated that central MC4 receptor activation leads to sympathoexcitation and a consequent increase in blood pressure [22–25]. Therefore, if the therapeutic potential of MC receptor targeted treatments is further addressed in experimental models of hypertension, it will be important to control and limit the MC4-R-mediated effects on hemodynamics by using compounds that do not cross the blood–brain-barrier or activate MC4 receptors to a significant extent.

In hypertension and many other important pathological conditions, the function of the NO-cGMP-axis is compromised leading to impaired endothelium-dependent control of vascular tone and elevated vascular resistance [26]. These features are also recapitulated in the DOCA-salt model of hypertension [27,28]. In line with this view, we found that urinary cGMP excretion in saline-treated DOCA-salt mice was suppressed to a level below the baseline, reflecting disturbed integrity of the NO-cGMP pathway. Based on our recent finding that α-MSH by acting through MC1 receptors enhances vascular NO availability and endothelial function [5], it is possible that the vascular effects of α-MSH have contributed to the antihypertensive potency of NDP-α-MSH treatment. It is particularly noteworthy that cGMP excretion was increased in NDP-α-MSH-treated mice, pointing to enhanced signaling through the NO-cGMP pathway. On the other hand, the heightened NO formation in DOCA-salt mice might be largely attributable to an increase in inducible NO synthase (iNOS)-derived NO rather than to an increase in endothelial (NOS) bioactivity [29]. Thus, although NO excretion was similarly increased in saline- and NDP-α-MSH-treated mice under DOCA-salt challenge, it does not solely represent the local availability of eNOS-derived NO in blood vessels which is critical for the maintenance of vascular homeostasis. Taken together, the disintegration of the NO-cGMP pathway represents a critical determinant of blood pressure development in DOCA-salt hypertension and NDP-α-MSH treatment may provide additional benefits in the management of hypertension through its positive impact on the eNOS system and NO-mediated vascular responses. Although the relative contributions of the natriuretic and vascular effects to the antihypertensive efficacy of α-MSH-treatment remain unknown, the underlying mechanism is probably multifactorial, involving influences on both blood vessels and renal function.

It is well-known that oxidative stress plays a crucial role in the pathogenesis of DOCA-salt hypertension [30,31]. Based on studies showing that α-MSH can suppress oxidative stress in various disease models [11,12], we expected to see an improvement in the redox balance of NDP-α-MSH-treated mice under DOCA-salt challenge. However, despite the increased urinary 8-isoprostane levels, DOCA-salt hypertension did not aggravate the oxidative stress at the tissue level as evidenced by the unaltered cardiac and renal CD levels and by the modest increase in vascular ROS production. Unexpectedly, the cardiac and renal 8-isoprostane levels were reduced in DOCA-salt mice. Considering that 8-isoprostane is primarily derived from the oxidation of arachidonic acid, chronic DOCA-salt challenge may lead to a depletion of the tissue reservoirs of arachidonic acid, thus limiting further formation of 8-isoprostane. Altogether, the data suggest that the 21-day DOCA-salt exposure, although enhancing oxidative processes, does not entail a generalized oxidative stress at the tissue level. It is possible that effective antioxidant systems in this mouse strain have maintained adequate redox balance in tissues under observation, which, in turn, may have prevented the appearance of possible antioxidant effects of NDP-α-MSH. Further research will be needed to assess whether α-MSH analogues, with appropriate dosage regimens in more severe models of hypertension, could provide protection against oxidative stress and associated cardiovascular pathology.

In conclusion, the present findings demonstrate that administration of NDP-α-MSH, a stable analogue of α-MSH, induces marked diuretic and natriuretic responses through the activation of MC3/4 receptor pathways without affecting renal potassium handling. These characteristics provided protection against hypernatremia and blood pressure elevation in the DOCA-salt model of hypertension, but other mechanisms such as promotion of vascular function via endothelial MC1 receptors are likely to contribute to the antihypertensive efficacy of NDP-α-MSH.

Melanocortins have been previously shown to participate in the regulation of sodium homeostasis and vascular function, but here we show for the first time that pharmacological targeting of melanocortin receptors with a pan-agonist, NDP-α-MSH, possesses therapeutic efficacy in experimental hypertension. Since sodium accumulation together with impaired vascular function contributes to the development of hypertension and associated cardiovascular pathologies, NDP-α-MSH treatment might have a dual therapeutic effect by interrupting these critical pathophysiological processes. The present data demonstrate that NDP-α-MSH could alleviate sodium retention in the DOCA-salt model of hypertension via MC3-R-mediated diuretic and natriuretic actions. Furthermore, our recent observation that α-MSH promotes vascular function via MC1 receptors might add therapeutic value for NDP-α-MSH treatment in the management of hypertension. These findings suggest applicability of α-MSH analogues for therapeutic use in hypertensive disease states and merit further attention to investigate whether selective and dual targeting of MC1 and MC3 receptors could have beneficial effects in more severe models of hypertension.

## Supporting Information

Figure S1 **Analysis of NDP-α-MSH-evoked weight reduction by quantitative NMR scanning.** Change in body weight (A), total body water (B), fat mass (C) and lean mass (D) after NDP-α-MSH administration. Body composition was analyzed by quantitative NMR scanning before and 2 hours after an i.p. injection of NDP-α-MSH (0.3 mg/kg). * *P* < 0.05 and ** *P* < 0.01 versus saline-treated mice. Data are mean ± SEM of six mice per group.
(TIF)

Figure S2 **ROS formation in the aorta.** (**A**) *In situ* dihydroethidium (DHE) staining of aortae from control and DOCA-salt mice. Red fluorescence indicates the presence of superoxide anions. Elastin fibers are seen as green due to autofluorescence. All sections are shown with the lumen at the right. Scale bars 100 μm. (**B**) Analysis of DHE intensity relative to saline-treated control mice. ## *P* < 0.01 versus control mice. Data are mean ± SEM of four mice per group.
(TIF)

Figure S3 **Oxidative stress markers in the heart and kidney.** Levels of 8-isoprostanes and conjugated dienes (CD) in the heart (**A**, **C**) and kidney (**B**, **D**). Number of mice analyzed is given in Table 1. # *P* < 0.05 versus control mice.
(TIF)

## References

[1] Orias R, McCann SM (1972) Natriuresis induced by alpha and beta melanocyte stimulating hormone (MSH) in rats. Endocrinology 90: 700-706. doi:10.1210/endo-90-3-700. PubMed: 5009346. 5009346 10.1210/endo-90-3-700

[2] Hradec J, Horký K (1979) Natriuretic and kaliuretic effect of melanocyte-stimulating hormones in hamsters. Endocrinol Exp 13: 145-152. PubMed: 315865. 315865

[3] Lymangrover JR, Buckalew VM, Harris J, Klein MC, Gruber KA (1985) Gamma-2MSH is natriuretic in the rat. Endocrinology 116: 1227-1229. doi:10.1210/endo-116-3-1227. PubMed: 3971908. 3971908 10.1210/endo-116-3-1227

[4] Humphreys MH (2004) Gamma-MSH, sodium metabolism, and salt-sensitive hypertension. Am J Physiol Regul Integr Comp Physiol 286: R417-R430. doi:10.1152/ajpregu.00365.2003. PubMed: 14761863. 14761863 10.1152/ajpregu.00365.2003

[5] Rinne P, Nordlund W, Heinonen I, Penttinen AM, Saraste A et al. (2013) α-Melanocyte-stimulating hormone regulates vascular NO availability and protects against endothelial dysfunction. Cardiovasc Res 97: 360-368. doi:10.1093/cvr/cvs335. PubMed: 23131503. 23131503 10.1093/cvr/cvs335 PMC3543993

[6] Giuliani D, Mioni C, Bazzani C, Zaffe D, Botticelli AR et al. (2007) Selective melanocortin MC4 receptor agonists reverse haemorrhagic shock and prevent multiple organ damage. Br J Pharmacol 150: 595-603. PubMed: 17245369. 17245369 10.1038/sj.bjp.0707115 PMC2189765

[7] Bazzani C, Guarini S, Botticelli AR, Zaffe D, Tomasi A et al. (2001) Protective effect of melanocortin peptides in rat myocardial ischemia. J Pharmacol Exp Ther 297: 1082-1087. PubMed: 11356932. 11356932

[8] Gatti S, Lonati C, Acerbi F, Sordi A, Leonardi P et al. (2012) Protective action of NDP-MSH in experimental subarachnoid hemorrhage. Exp Neurol 234: 230-238. doi:10.1016/j.expneurol.2011.12.039. PubMed: 22230666. 22230666 10.1016/j.expneurol.2011.12.039

[9] Touyz RM, Briones AM (2011) Reactive oxygen species and vascular biology: implications in human hypertension. Hypertens Res 34: 5-14. doi:10.1038/hr.2010.201. PubMed: 20981034. 20981034 10.1038/hr.2010.201

[10] Colombo G, Gatti S, Turcatti F, Sordi A, Fassati LR et al. (2005) Gene expression profiling reveals multiple protective influences of the peptide alpha-melanocyte-stimulating hormone in experimental heart transplantation. J Immunol 175: 3391-3401. PubMed: 16116233. 16116233 10.4049/jimmunol.175.5.3391

[11] Kolgazi M, Arbak S, Alican I (2007) The effect of alpha-melanocyte stimulating hormone on gentamicin-induced acute nephrotoxicity in rats. J Appl Toxicol 27: 183-188. doi:10.1002/jat.1191. PubMed: 17216604. 17216604 10.1002/jat.1191

[12] Lindskog A, Ebefors K, Johansson ME, Stefánsson B, Granqvist A et al. (2010) Melanocortin 1 receptor agonists reduce proteinuria. J Am Soc Nephrol 21: 1290-1298. doi:10.1681/ASN.2009101025. PubMed: 20507942. 20507942 10.1681/ASN.2009101025 PMC2938589

[13] Rinne P, Harjunpää J, Scheinin M, Savontaus E (2008) Blood pressure regulation and cardiac autonomic control in mice overexpressing alpha- and gamma-melanocyte stimulating hormone. Peptides 29: 1943-1952. doi:10.1016/j.peptides.2008.06.012. PubMed: 18638516. 18638516 10.1016/j.peptides.2008.06.012

[14] Ruohonen ST, Savontaus E, Rinne P, Rosmaninho-Salgado J, Cavadas C et al. (2009) Stress-induced hypertension and increased sympathetic activity in mice overexpressing neuropeptide Y in noradrenergic neurons. Neuroendocrinology 89: 351-360. doi:10.1159/000188602. PubMed: 19122447. 19122447 10.1159/000188602

[15] Kathpalia PP, Charlton C, Rajagopal M, Pao AC (2011) The natriuretic mechanism of Gamma-Melanocyte-Stimulating Hormone. Peptides 32: 1068-1072. doi:10.1016/j.peptides.2011.02.006. PubMed: 21335042. 21335042 10.1016/j.peptides.2011.02.006 PMC3112371

[16] Cope G, Flanagan ET, Houghton BL, Walsh SA, Johns EJ et al. (2012) The analogue, NDP-γ(2) MSH, possesses the renal excretory but not the cardiovascular actions of the native peptide, gamma2-melanocyte stimulating hormone in anaesthetized rats. Clin Exp Pharmacol Physiol. 10.1111/1440-1681.12025 23106106

[17] Ni XP, Bhargava A, Pearce D, Humphreys MH (2006) Modulation by dietary sodium intake of melanocortin 3 receptor mRNA and protein abundance in the rat kidney. Am J Physiol Regul Integr Comp Physiol 290: R560-R567. PubMed: 16195498. 16195498 10.1152/ajpregu.00279.2005

[18] Ni XP, Kesterson RA, Sharma SD, Hruby VJ, Cone RD et al. (1998) Prevention of reflex natriuresis after acute unilateral nephrectomy by melanocortin receptor antagonists. Am J Physiol 274: R931-R938. PubMed: 9575953. 9575953 10.1152/ajpregu.1998.274.4.R931

[19] Chen XW, Ying WZ, Valentin JP, Ling KT, Lin SY et al. (1997) Mechanism of the natriuretic action of gamma-melanocyte-stimulating hormone. Am J Physiol 272: R1946-R1953. PubMed: 9227612. 9227612 10.1152/ajpregu.1997.272.6.R1946

[20] Schenk J, McNeill JH (1992) The pathogenesis of DOCA-salt hypertension. J Pharmacol Toxicol Methods 27: 161-170. doi:10.1016/1056-8719(92)90036-Z. PubMed: 1498343. 1498343 10.1016/1056-8719(92)90036-z

[21] Ugwu SO, Blanchard J, Dorr RT, Levine N, Brooks C et al. (1997) Skin pigmentation and pharmacokinetics of melanotan-I in humans. Biopharm Drug Dispos 18: 259-269. doi:10.1002/(SICI)1099-081X(199704)18:3. PubMed: 9113347. 9113347 10.1002/(sici)1099-081x(199704)18:3<259::aid-bdd20>3.0.co;2-x

[22] Greenfield JR, Miller JW, Keogh JM, Henning E, Satterwhite JH et al. (2009) Modulation of blood pressure by central melanocortinergic pathways. N Engl J Med 360: 44-52. doi:10.1056/NEJMoa0803085. PubMed: 19092146. 19092146 10.1056/NEJMoa0803085

[23] Kuo JJ, da Silva AA, Tallam LS, Hall JE (2004) Role of adrenergic activity in pressor responses to chronic melanocortin receptor activation. Hypertension 43: 370-375. doi:10.1161/01.HYP.0000111836.54204.93. PubMed: 14707160. 14707160 10.1161/01.HYP.0000111836.54204.93

[24] Ni XP, Butler AA, Cone RD, Humphreys MH (2006) Central receptors mediating the cardiovascular actions of melanocyte stimulating hormones. J Hypertens 24: 2239-2246. doi:10.1097/01.hjh.0000249702.49854.fa. PubMed: 17053546. 17053546 10.1097/01.hjh.0000249702.49854.fa

[25] Matsumura K, Tsuchihashi T, Abe I, Iida M (2002) Central alpha-melanocyte-stimulating hormone acts at melanocortin-4 receptor to activate sympathetic nervous system in conscious rabbits. Brain Res 948: 145-148. doi:10.1016/S0006-8993(02)03045-7. PubMed: 12383966. 12383966 10.1016/s0006-8993(02)03045-7

[26] Förstermann U, Li H (2011) Therapeutic effect of enhancing endothelial nitric oxide synthase (eNOS) expression and preventing eNOS uncoupling. Br J Pharmacol 164: 213-223. doi:10.1111/j.1476-5381.2010.01196.x. PubMed: 21198553. 21198553 10.1111/j.1476-5381.2010.01196.x PMC3174401

[27] Van de Voorde J, Leusen I (1986) Endothelium-dependent and independent relaxation of aortic rings from hypertensive rats. Am J Physiol 250: H711-H717. PubMed: 3458379. 3458379 10.1152/ajpheart.1986.250.5.H711

[28] Shirasaki Y, Kolm P, Nickols GA, Lee TJ (1988) Endothelial regulation of cyclic GMP and vascular responses in hypertension. J Pharmacol Exp Ther 245: 53-58. PubMed: 2834546. 2834546

[29] Obst M, Gross V, Bonartsev A, Janke J, Müller DN et al. (2004) Nitric oxide synthase expression in AT2 receptor-deficient mice after DOCA-salt. Kidney Int 65: 2268-2278. doi:10.1111/j.1523-1755.2004.00646.x. PubMed: 15149340. 15149340 10.1111/j.1523-1755.2004.00646.x

[30] Manning RD, Meng S, Tian N (2003) Renal and vascular oxidative stress and salt-sensitivity of arterial pressure. Acta Physiol Scand 179: 243-250. doi:10.1046/j.0001-6772.2003.01204.x. PubMed: 14616240. 14616240 10.1046/j.0001-6772.2003.01204.x

[31] Beswick RA, Dorrance AM, Leite R, Webb RC (2001) NADH/NADPH oxidase and enhanced superoxide production in the mineralocorticoid hypertensive rat. Hypertension 38: 1107-1111. doi:10.1161/hy1101.093423. PubMed: 11711506. 11711506 10.1161/hy1101.093423

