# Supplementary figures and images for: α-MSH Analogue Attenuates Blood Pressure Elevation in DOCA-Salt Hypertensive Mice
Source: PLoS One. 2013 Aug 16;8(8):e72857. doi: 10.1371/journal.pone.0072857 (PMC3745458; doi:10.1371/journal.pone.0072857)

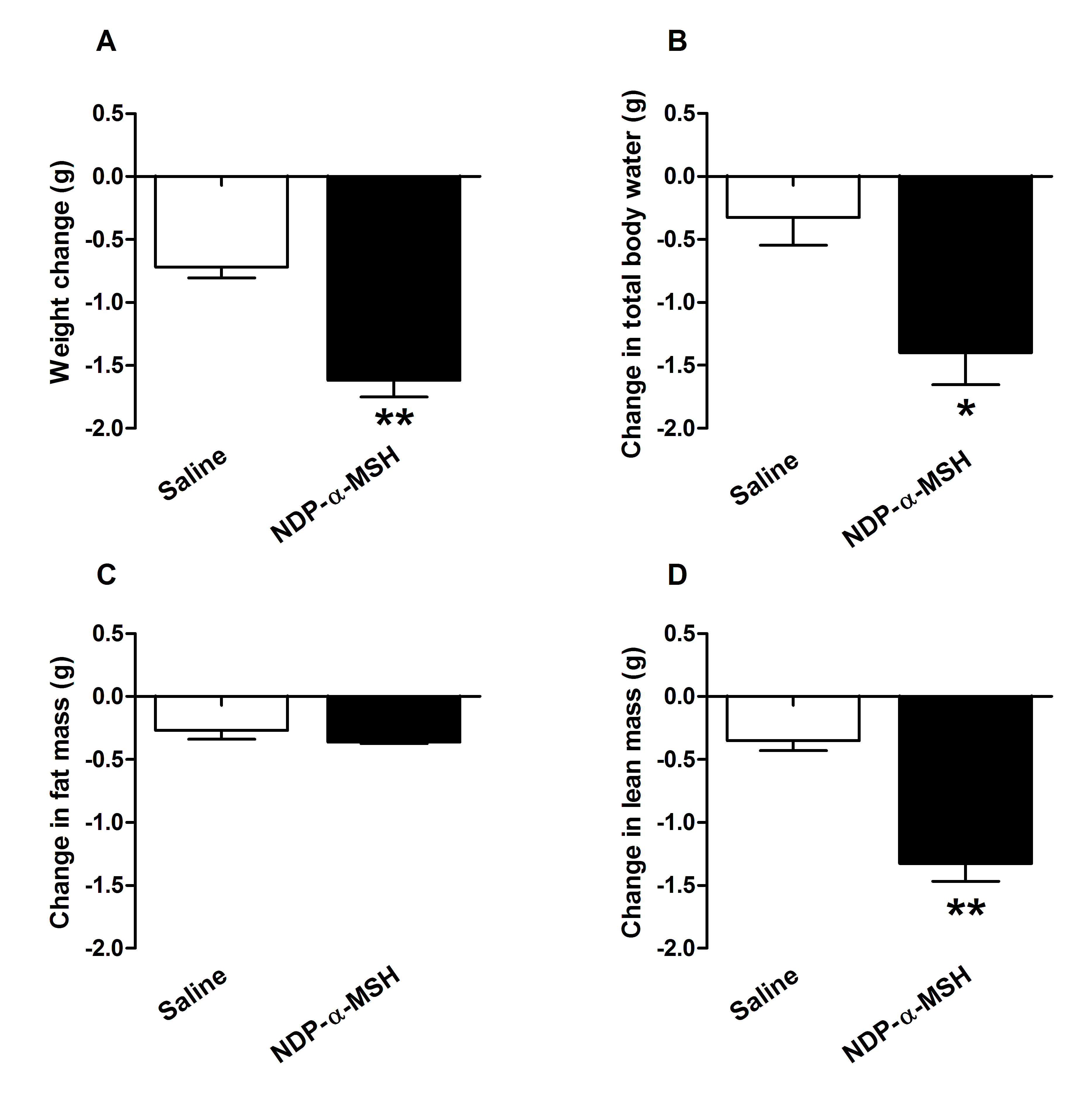

Supplement: Figure S1 — Analysis of NDP-α-MSH-evoked weight reduction by quantitative NMR scanning. Change in body weight (A), total body water (B), fat mass (C) and lean mass (D) after NDP-α-MSH administration. Body composition was analyzed by quantitative NMR scanning before and 2 hours after an i.p. injection of NDP-α-MSH (0.3 mg/kg). * P < 0.05 and ** P < 0.01 versus saline-treated mice. Data are mean ± SEM of six mice per group. (TIF) [file pone.0072857.s001.tif]

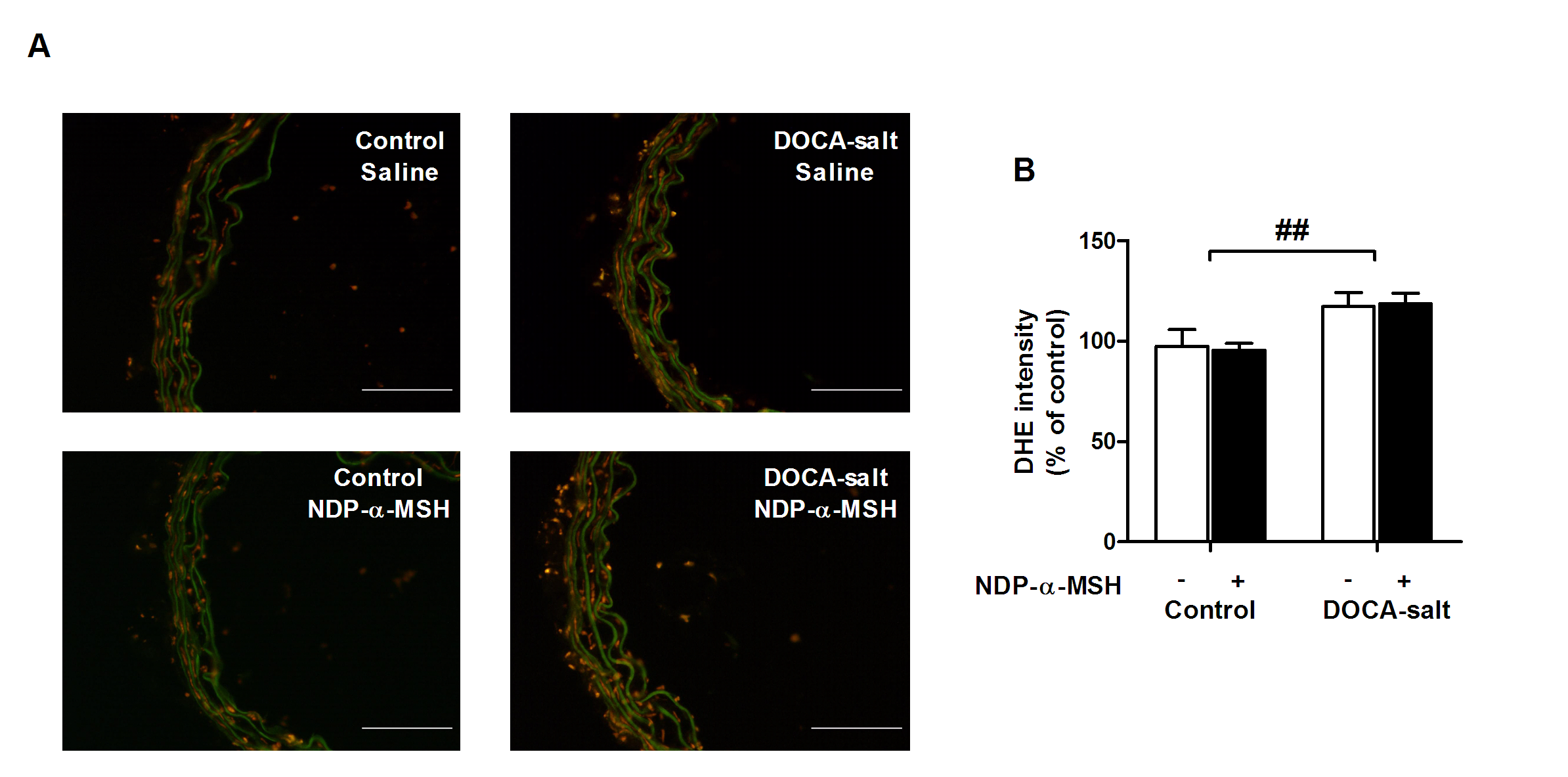

Supplement: Figure S2 — ROS formation in the aorta. (A) In situ dihydroethidium (DHE) staining of aortae from control and DOCA-salt mice. Red fluorescence indicates the presence of superoxide anions. Elastin fibers are seen as green due to autofluorescence. All sections are shown with the lumen at the right. Scale bars 100 μm. (B) Analysis of DHE intensity relative to saline-treated control mice. ## P < 0.01 versus control mice. Data are mean ± SEM of four mice per group. (TIF) [file pone.0072857.s002.tif]

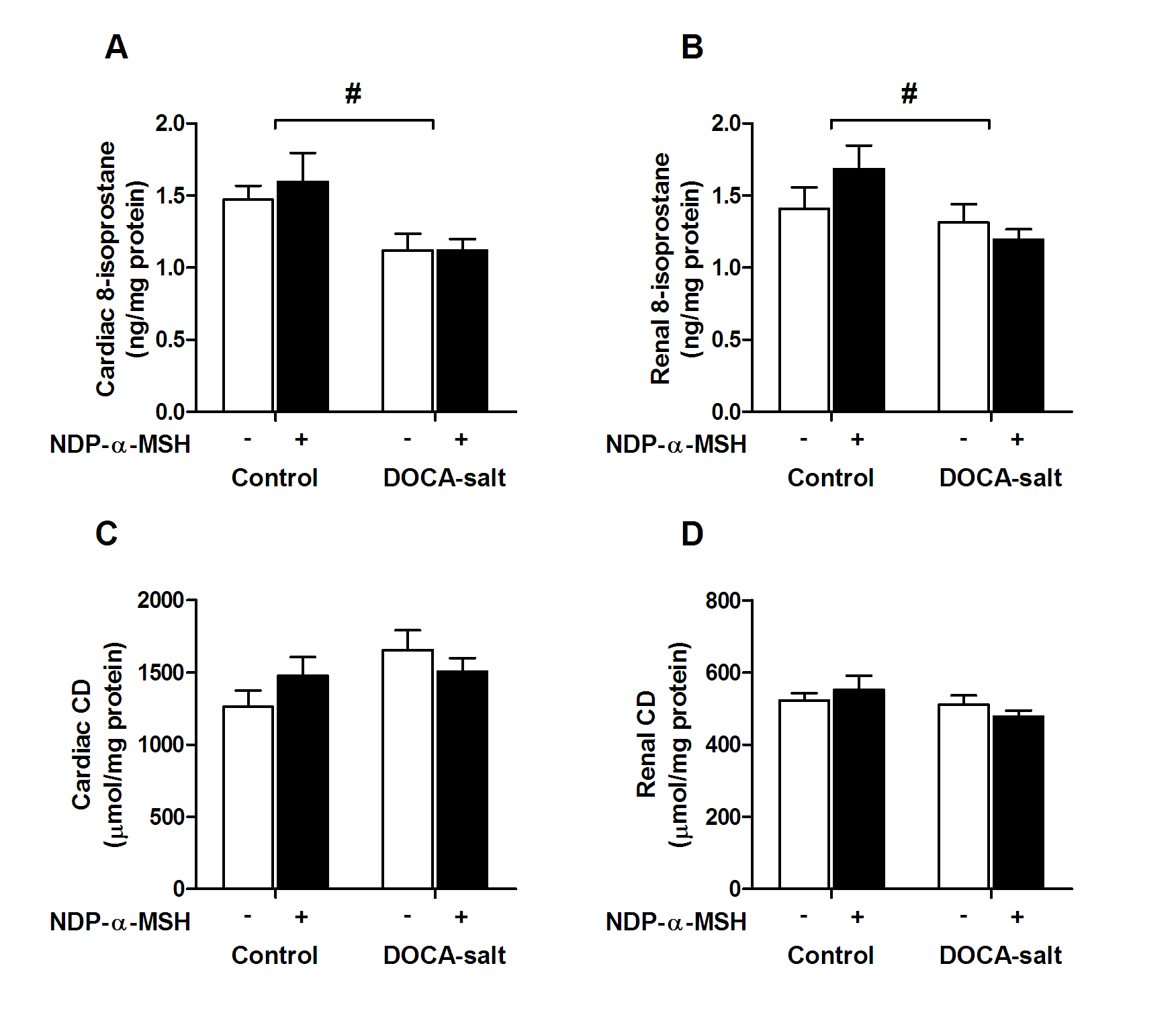

Supplement: Figure S3 — Oxidative stress markers in the heart and kidney. Levels of 8-isoprostanes and conjugated dienes (CD) in the heart (A, C) and kidney (B, D). Number of mice analyzed is given in Table 1. # P < 0.05 versus control mice. (TIF) [file pone.0072857.s003.tif]
